# Privatization of Medical Services and Revenue Development Project: A Cross-Sectional Survey of Staff Perceptions at the University of Jeddah Medical Center

**DOI:** 10.3390/healthcare11182540

**Published:** 2023-09-14

**Authors:** Mansour Tobaiqy, Ahlam Alrefai, Mohammed Esmail Qashqary, Rashed Al Sulami, Shrooq T. Aldahery

**Affiliations:** 1 Department of Pharmacology, College of Medicine, University of Jeddah, Jeddah P.O. Box 45311, Saudi Arabia; 2 Medical Services Administration, University of Jeddah, Jeddah P.O. Box 45311, Saudi Arabia; asaljuhani@uj.edu.sa (A.A.); rralsulami@uj.edu.sa (R.A.S.); 3 Department of Family and Community Medicine, College of Medicine, University of Jeddah, Jeddah P.O. Box 45311, Saudi Arabia; meqashqary@uj.edu.sa; 4 Department of Applied Radiologic Technology, College of Applied Medical Sciences, University of Jeddah, Jeddah P.O. Box 23817, Saudi Arabia; staldahery@uj.edu.sa

**Keywords:** medical service, paid treatment, privatization, revenue

## Abstract

This study aimed to assess the perceptions of staff working at the University of Jeddah (UJ) Medical Center on the possibility of finding new financing methods for the administration and privatization of the primary and specialized medical care services it provides. A questionnaire link was sent online targeting all staff at the UJ Medical Center (n = 141). The questionnaire comprised 17 items under the following sections: demographic information, staff perceptions about the current status of the services provided by the UJ Medical Center and the possibility of finding new financing methods and additional sources of revenue for the administration. Of the 101 questionnaires returned, the majority were filled by males (n = 71; 70.3%). One-third of the participants (n = 39; 38.6%) have between 5 and 9 years of working experience in Medical Administration, and most of them (n = 42; 41.6%) reported that they have a background in the concept of revenue development/privatization/self-resources/paid treatment. Most were satisfied with the current status of the services provided (average rating = 3.39/5). However, most participants (n = 72; 71.3%) reported that the UJ Medical Center is not ready for the Revenue Development Project of privatization. The survey respondents demonstrated satisfaction with the medical services provided by the UJ Medical Center and the potential application of the Revenue Development Project. However, streamlining the privatization process according to the governmental structures is crucial for it to be implemented properly at the UJ Medical Center.

## 1. Introduction

The Saudi Arabian public health service has a long and storied history [1]. The first public health department was created in 1925 by King Abdul Aziz [2]. This department was responsible for providing essential healthcare services to the population, such as vaccination, sanitation, and disease prevention. In the 1950s, the Saudi Arabian government began to invest heavily in the healthcare sector [2]. This investment led to a significant improvement in the quality and availability of healthcare services. By the 1970s, Saudi Arabia had a well-developed public health system comparable to developed countries [1,2]. The Saudi Arabian population is growing rapidly [3], and it is estimated that it will reach 55 million by 2050 [3]. This growth will put a strain on the country’s healthcare system. The government is working to expand the healthcare system to meet the needs of the growing population [3]. The Ministry of Health (MOH) manages the Saudi Arabian healthcare system [1,2,3], and is responsible for providing healthcare services to all citizens and residents of Saudi Arabia. The Ministry of Health operates a network of hospitals, clinics, and primary healthcare centers. There are currently 268 hospitals in Saudi Arabia [4], which provide a wide range of healthcare services, including inpatient, outpatient, and emergency care. The hospitals are equipped with the latest medical technology and staffed by highly qualified healthcare professionals [2,4].

The Saudi Arabian vision 2030 highlights the role of the private sector as the main engine for social and economic reform. It also emphasizes fostering private sector investment in sectors previously funded by the government [1,5,6]. It is expected that the private sector contributes to sustainable development by bringing both the public and private sectors together. This connection helps to improve the level of care, increase patient satisfaction, and reduce financial risks in the public sector [1,5].

The privatization initiative in the medical healthcare setting in Saudi Arabia presents both challenges and opportunities. According to Alkhamis et al., cost containment is a significant influencer of health restructuring worldwide, and governments, including that of Saudi Arabia, are under pressure to build sustainable health models [5]. Public–Private Partnerships (PPPs) were proposed to deliver health services, aiming to transition from the traditional regulator-pay-or-deliver model to a more sustainable model. However, this transition has its challenges [5,6,7,8]. Some challenges faced by the healthcare system in Saudi Arabia include inefficiencies built into the system, such as duplication of services, lack of coordination between different stakeholders, and overstaffing. In addition, unhealthy lifestyles lead to morbidities in a large section of the population, rising healthcare costs, and premature deaths [5,6,7,8]. Despite these challenges, the MOH plans to develop more inclusive and sustainable PPP models. This presents opportunities for private players to participate in the healthcare sector and contribute to its growth [5].

There is no doubt that development plans have had major success in creating the infrastructure needed to offer treatment services in Saudi Arabia, and the fields of medical education and training for technical staff in the health profession have seen extraordinary advancement [1]. The importance of primary healthcare (PHC) in health systems has also been acknowledged [9,10]. Based on different services and health needs, PHC services are provided for various conditions to ensure that society can access integrated and comprehensive healthcare services [10,11,12]. High-quality public healthcare services are introduced in all governmental universities in Saudi Arabia and provide comprehensive preventive and therapeutic services to students and staff throughout the year [11,12,13].

Medical centers are considered one of the most important sectors in universities due to the need to provide healthcare to all students and employees of the university and those who are eligible for treatment [14,15]. PHC has proven to be an effective and efficient means of addressing health’s main causes and risks, improving health security to prevent and respond to acute threats such as epidemics and antimicrobial resistance [14,15]. The university medical centers meet the needs of the university’s students and employees in terms of prevention and treatment. They achieve this through specialized clinics and supportive medical services that contribute to the integration of medical care provided with the capabilities available to it and under the quality standards of health services, to improve the level of performance and gain the satisfaction of the service beneficiaries [16,17].

Of note, the privatization of medical services at universities in Saudi Arabia is still in its early stages [6,7,8]. However, it has been successfully implemented in some MOH healthcare facilities to increase self-resources, and as a part of the PPP strategy, where the private sector was allowed to build and operate new hospitals and medical centers. These PPPs allow the government to share construction and operation costs with private investors while ensuring the facilities are accessible to all citizens [7].

In line with the privatization of medical services in Saudi Arabia, Wasfaty is an e-prescribing service, and a new digital transformation initiative recently introduced. It aims to transfer pharmaceutical care services from primary healthcare centers to community pharmacies. The MOH implemented this service as part of its measures to facilitate medication dispensing services and reduce costs [18]. Moreover, some universities’ medical centers have also implemented Wasfaty prescription and medicine dispensing services [18].

The privatization of medical services at universities in Saudi Arabia has several potential benefits [7,8,19,20,21]. For example, it could lead to improved quality of care, as private providers are often more motivated to provide high-quality services. It could also increase efficiency as private providers are often more cost-effective than public providers. However, there are also some potential drawbacks; privatization could increase patient costs, as private providers are often more expensive than public providers. It could also lead to decreased access to care, as private providers may only be willing to serve some patients, especially those uninsured or underinsured [7,8,19,20,21].

These considerations suggest that funding PHC should be a major priority, particularly considering the evidence that it is essential for improving population health and minimizing medical expenses [21]. However, PHC accessibility and services remain a concern for the general public, even in countries with outstanding healthcare systems, such as the United Kingdom, India, China, and Australia [20]. Prudent investment and the growth of PHC revenues are two pillars of strategic development and improvement of services; hence, it is necessary to raise and improve the level of PHC performance and fulfil its goals. Therefore, this study aimed to assess staff satisfaction about the services provided by the Medical Center and to assess perspectives on the privatization of primary and specialized medical care services provided by the Medical Center at the University of Jeddah.

## 2. Methods

### 2.1. Study Design

The research design was a cross-sectional survey of all staff that work at the Medical Center (n = 142), University of Jeddah (UJ), Jeddah, Saudi Arabia.

### 2.2. Questionnaire Development

The questionnaire was designed and built through the application of brainstorming sessions and panel discussion among the research team., Its design involved multiple steps drafting, content-focused and data-focused pilots, literature reviews, and careful consideration of outcomes to measure. The questionnaire contained 17 questions comprising items of demographics, education and health specialty, staff perceptions around the current status of the medical services provided by the UJ Medical Center and the privatization of medical services, and their opinions whether the Revenue Development Project (RDP) has been implemented to provide medical services for a fee. The aim was to assess staff satisfaction about the services provided by the medical center and staff perspectives on the ways of generating revenue for the primary and specialized medical care services provided. Item types included open-ended, closed, and 5-point Likert scale questions. Some questionnaire items were formulated so as to allow respondents to express their opinions or experiences in their own words, while other questions offered predefined choices or scales to select from.

Before use, the research team reviewed the questionnaire for face and content validity, which included three academic staff at the Colleges of Medicine and Applied Medical Sciences and two healthcare administrative staff.

Validity testing was followed by piloting the questionnaire using web-based Microsoft Forms to five staff of UJ Medical Center between April and June 2023, using a non-probability convenient sampling technique [22]. As some changes were made to the questionnaire post piloting, the data generated by the pilot study were not included in the main study dataset.

### 2.3. Recruitment

Potential participants were approached by personalized invitation using university e-mail. Participants had to be staff at the UJ Medical Center; there were no exclusion criteria. Each potential participant was provided with a study information leaflet, and those who agreed to participate were asked to complete the survey electronically.

### 2.4. Statistical Analysis

The data was analyzed using the SPSS statistical package (IBM SPSS Statistics version 29.0, PASW, Chicago, IL, USA). Descriptive statistics, including frequencies, ratios, and rates, were used in the analysis. Pearson’s chi-squared test was also used to study the relationship between variables and to determine any association between the expected improvement in medical services when implementing the Revenue Development Project (the main outcome) and each independent variable. A *p*-value of less than 0.05 was considered statistically significant.

## 3. Results

One hundred and one participants completed the questionnaires giving a response rate of 71% (101/142); the majority were males (n = 71; 70.3%). One-third were educated with a bachelor’s degree (n = 34; 33.7%), over one-third possessed an undergraduate diploma degree (n = 36; 35.6%), less than one-third held an MD or PhD degree (n = 24; 23.8%), and the rest held a master’s degree (n = 7; 6.9%). The majority of participants were technicians (n = 42), physicians (n = 28), and health specialists (n = 17). The majority were in the age group between 30 and 39 years (n = 67; 66.3%), and the minority were in the age group of less than 30 years (n = 5; 5%) (Table 1).

### 3.1. Respondents’ Satisfaction with Medical Services and Medical Services for Potential Improvement

The majority were satisfied with the medical services provided by the Medical Center; their responses were ‘Very Satisfied’ (n = 20; 19.8%) and ‘Satisfied’ (n = 35; 34.7%), However, for less than one-third, the response was ‘Neutral’ (n = 25; 24.8%). There was an equal number of responses concerning the possible opportunities for improvement of medical services, where the majority indicated that infrastructure (n = 62; 61.3%), technical infrastructure (n = 60; 59.4%), supporting medical services (pharmacy, laboratory, radiology, vaccination unit, nursing care, optics) and health education (n = 59; 58.4%), and logistics and supply services (n = 57; 56.4%) were the services that most needed improvement. The other half of their responses indicated that the following aspects needed to be improved: empowerment of employees, training programs, communication, and self-management (n = 51; 50.5%). Over one-third responded that the organization aspects within the Medical Center (n = 40; 39.6%) and primary medical care (clinics) (n = 37; 36.6%) needed improvement (Table 2).

### 3.2. Responses on Revenue Development for the Medical Center Services

The majority of respondents are familiar with the terms revenue development, privatization, self-resources, paid treatment, and business center (n = 42; 41.6%), while one-third answered that they had no background on the concept (n = 31; 30.7%). Less than one-third responded “I am not sure” (n = 28; 27.7%), and the majority of them work in the technical field (Table 2).

The majority of respondents did not believe that the Medical Center is ready to provide its medical services for a fee (n = 72; 71.3%) (Table 3). That may be linked to the respondents’ previous reports that the Medical Center has various deficiencies in the medical services it provided (Table 2). This suggests that the Medical Center administration will witness multiple challenges when implementing the Revenue Development Project. However, over half of the respondents (n = 57; 56.4%) agreed that applying the Revenue Development Project will work effectively; the majority of them have over 5 years of working experience in the Medical Center. In contrast, the least frequent answer was ‘Disagree’ (n = 7; 6.9%).

The responses around the potential services in the Medical Center that will represent sustainable revenue when provided for a fee was of an equal number (Table 3).

### 3.3. Areas for Potential Revenue of Medical Services

The majority responded that implementing the role of virtual clinics for all medical clinics would be a potential revenue source for the Medical Center (n = 58; 57.4%), together with the use of primary medical services (clinical examination, laboratory, radiology) in joint projects with other sectors (n = 57; 56.4%). According to the respondents, another promising area for potential revenue was organizing scientific conferences and training courses (n = 52; 51.4%) and investing in unused space for a fee (n = 52; 51.4%). On the other hand, over one-third (n = 49; 48.5%) thought that medical research, scientific papers, and providing scientific consulting services to companies, individuals, and various sectors of society would be a potential benefit for the Medical Center. The majority of respondents believed that the Revenue Development Project would contribute to increasing the income of the employees of the Medical Center and motivating them (n = 60; 59.4%), and that it would contribute to achieving cash savings to manage medical services and bridge the budget deficit (n = 65; 64.3%) (Table 3). The majority of respondents in Leadership and Supervisory roles in the Medical Center agreed with that statement.

The survey found that there was no significant association between the perceptions for staff on the extent of the expected improvement in medical services when implementing the Revenue Development Project and their working position (*p* = 0.855), educational level (*p* = 0.122), or working experience (*p* = 0.017).

### 3.4. Comments and Suggestions of Respondents

Respondents have provided suggestions and comments regarding the areas of the medical services that need to be developed and improved, as well as challenges that the Medical Center may encounter in developing this project, as presented in Table 4.

## 4. Discussion

The current study has evaluated the perspectives of the Medical Center’s staff on the privatization of primary and specialized medical care services. While the majority were satisfied or very satisfied with the medical services provided by the UJ Medical Center and the potential application of the Revenue Development Project, the majority also believed that the UJ Medical Center is not ready for the Revenue Development Project at the current time. The respondents reported that one of the most important issues that impede the implementation of such projects is the lack of medical professionals in medical centers [23]. They also believed that virtual clinics, using primary medical services (clinical examinations, laboratory, radiology), organizing scientific conferences and training courses, and offering consulting services to companies would be a potential benefit for the medical centers and an area of privatization of services. Of note, respondents believed that the Revenue Development Project would contribute to increasing the employees’ income and motivating them. It was reported by Rahman (2020), that privatizing health services would increase their effectiveness, quality, and public satisfaction while enabling the government to perform its constitutional obligations [8]. A study was conducted by Al-Mubarak et al. (2021), to investigate different healthcare professionals’ insights about privatization of the Saudi healthcare sectors as they found that conflicting governance structures, and inadequate and unclear communication hindered the plan’s execution. However, it would give it a chance to compete with private sectors [24]. The improvement of economic enablers by fostering the expansion of the private sector is one of the Vision 2030 pillars, which can be examined in light of these findings.

In addition, the results of this study revealed the participants’ suggestions of how to privatize various medical services that can be provided particularly at university medical centers, which would significantly increase the ability to create PPPs in the Saudi healthcare sector.

In this study, the majority of staff at the UJ Medical Center believe that the ancillary medical services, such as pharmacy, laboratory, radiology, and health education, are areas that can be improved. Tobaiqy et al. (2023) assessed the recently implemented e-prescribing and dispensing service at the UJ Medical Center, Wasfaty. They highlighted issues related to medicine availability and access to essential medicines [18]. As Aljuaid et al. (2016) argued, there is a growing demand for further improvement in healthcare quality at university medical centers to meet patients’ needs, including their satisfaction [4,18]. Therefore, it is essential to create coordinated, patient-centered health services that are of high quality, thus also allowing university medical centers to enhance their services competently and meet the community’s needs. Furthermore, one of the challenges that may constrain the provision of better healthcare and the improvement of healthcare quality at university medical centers is the need for more qualified healthcare professionals. Gamaleldeen (2015) highlighted that the diversity of nationalities working in the Saudi public health system, and the underutilization and inequity in resource distribution are some of the main challenges Saudi Arabia will face in the coming years [25]. Additionally, a study by Safi (2016) evaluated the difficulties that the Saudi healthcare system is experiencing, such as the underutilization and inequity in resource distribution [26]. The Ministry of Health (MOH) is the leading government provider and financier of healthcare services, accounting for 60% of all healthcare services in Saudi Arabia. The private sector, on the other hand, accounts for 27% of Saudi healthcare [26]. Therefore, the government promotes more private sector participation by providing long-term, interest-free financing to construct hospitals, clinics, and pharmacies. Thus, the privatization of services would help to mitigate the constraints identified by the participants in this study.

To the best of the authors’ knowledge, this is the first study to explore the potential of finding new sources of funding for medical center administrations as well as additional income streams to deliver paid healthcare in order to attain financial stability for the administration. All study participants were working at medical centers affiliated with different universities. The findings of this study showed that there are some medical services that can be privatized at university medical centers, such as activating laboratory services such as premarital examination, issuance of licenses, and examination of expatriate workers; providing training and scientific courses by the employees; and activating the role of virtual clinics. However, it was reported that there are some challenges to achieve these opportunities as the infrastructure services of the university medical centers are often inadequate to meet the patients’ demands. Furthermore, the lack of budget was reported as one of the most important factors that can lead to a restriction in the delivery of healthcare services. In Saudi Arabia, university medical centers are becoming recognized to deliver healthcare services to the universities’ employees. However, there is a need to raise the standard of healthcare delivery, particularly in the areas of patient safety and clinical efficacy [1].

The universities’ employees are used to receiving free services, including medical care. The results demonstrated that less than 50% (n = 35) of the study participants were satisfied with the administration’s services in the current situation. Therefore, there is a need to improve the logistics and strategies of the universities’ medical centers administration that can impact on healthcare service delivery. Technical assistance is essential to maintaining data security and patients’ privacy by offering all users the highest level of information protection and security [22]. Therefore, one of the most important keys to healthcare improvement as demonstrated by the study participants is to raise the quality of infrastructure and safety requirements. This was in line with a study by Luxon (2015) who reported that it can be achieved by incorporating information technology, architecture, design, commissioning a new hospital, and sustainability [27]. In addition, training, performance evaluation, and organizational development efforts can be used to raise staff clinical practice performance. A study by Potnuru et al. (2018) reported that an organization should foster a culture of learning that leads the staff members to exchange expertise, build teamwork, learn new clinical information, and develop skills that will develop creativity in the medical practice, which eventually can impact positively on employees’ competencies [28]. This may lead to questions on whether such logistics are applicable to the University of Jeddah Medical Services Administration, which ultimately follows the National Transformation Program, which attempts to build the required infrastructure and establish a climate that enables the public, private, and non-profit sectors to meet Vision 2030 needs [29].

### Limitations of the Study

While this study has added to the literature on the privatization of health care, some limitations may reduce the generalizability of the findings, including recruitment biases (the participants were from a single medical center and may not have been representative of all medical centers in the universities).

## 5. Conclusions

The majority of respondents in this study were satisfied with the medical services provided by the Medical Center and the potential application of the Revenue Development Project. However, the majority also believed that the UJ Medical Center is still being prepared for the Revenue Development Project at the current time. Future work should focus on how responsibility is applied and upheld in environments where it has never been performed. In addition, further research is required based on an upper-level administrative perspective, which can add huge value by providing a deeper understanding of the governmental structures to implement privatization properly in the future.

## Figures and Tables

**Table 1:** Demographic profile of the Medical Center staff over the study period (n = 101).

Variables	N	%
**Gender**		
Female	30	29.7
Male	71	70.3
**Age group**		
<30 years old	5	5.0
30–39 years	67	66.3
40–49 years	22	21.8
>50 years	7	6.9
**Educational level**		
Diploma	36	35.6
Bachelor’s degree	34	33.7
Master’s degree or equivalent	7	6.9
MD, PhD degree or equivalent	24	23.8
**Job classification**		
Physician	28	27.7
Health Specialist	17	16.8
Pharmacist	6	5.9
Technician	42	41.6
Administrative Personnel	8	7.9
**Working position**		
Leadership and Supervisory function	34	33.6
Technical function	58	57.4
Administrative function	13	12.8
other	11	10.8
**Working experience**		
0–3 years	29	28.7
5–9 years	39	38.6
<10 years	33	32.7

**Table 2:** Perceptions of UJ Medical Center staff on improvement of medical services.

Survey Questions	N	%
**Are you familiar with the following terms: revenue development/privatization/self-resources/paid treatment/business center?**		
Yes	42	41.6
No	31	30.7
I’m not sure	28	27.7
**How do you assess your satisfaction in general with the medical services of the UJ medical center in the current situation?**		
Very Satisfied	20	19.8
Satisfied	35	34.7
Neutral	25	24.8
Unsatisfied	17	16.8
Very Dissatisfied	4	4.0
**Which areas have opportunities for improvement and possible action to improve medical services?**		
Logistics and supply services	57	56.4
Infrastructure	62	61.3
Technical Infrastructure	60	59.4
Core competencies for employees (empowerment of employees, training programs, communication, self-management)	51	50.5
Primary medical care (clinics)	37	36.6
Supporting medical services (pharmacy, laboratory, radiology, vaccination unit, nursing care, optics) and health education	59	58.4
The organization aspects of the medical center	40	39.6
**Others**	4	3.9

**Table 3:** Staff perceptions about ways of generating revenue for the administration.

Survey Questions	N	%
**Is the UJ Medical Centre ready to provide its services for a fee?**		
Yes	29	28.7
No	72	71.3
**The application of the revenue development project will work effectively in the UJ Medical Centre**		
Strongly Agree	24	23.8
Agree	33	32.7
Neutral	30	29.7
Disagree	7	6.9
Strongly Disagree	7	6.9
**There will be an expected improvement in medical performance in the services subject to a paid treatment program**		
Strongly Agree	26	25.7
Agree	32	31.7
Neutral	33	32.7
Disagree	4	4.0
Strongly Disagree	6	5.9
**The revenue development project will increase efficiency and improve the services provided**		
Strongly Agree	31	30.7
Agree	34	33.7
Neutral	27	26.7
Disagree	3	3.0
Strongly Disagree	6	5.9
**Implementing the Revenue Development Project/a paid treatment program will contribute to achieving cash savings to manage medical services and bridge the budget deficit**		
Strongly Agree	29	28.7
Agree	36	35.6
Neutral	28	27.7
Disagree	2	2.0
Strongly Disagree	6	5.9
**The paid treatment program will contribute to developing and modernizing medical devices and equipment in the Medical Centre.**		
Strongly Agree	29	28.7
Agree	40	39.6
Neutral	22	21.8
Disagree	5	5.0
Strongly Disagree	5	5.0
**The implementation of the Revenue Development Project/a paid treatment program will contribute to the achievement of job satisfaction for the workers.**		
Strongly Agree	24	23.8
Agree	31	30.7
Neutral	33	32.7
Disagree	6	5.9
Strongly Disagree	7	6.9
**The revenue development project will contribute to increasing the income of the employees of the Medical Centre and motivating them.**		
Strongly Agree	29	28.7
Agree	31	30.7
Neutral	30	29.7
Disagree	6	5.9
Strongly Disagree	5	5.0
**Which services will represent an added value and sustainable revenue for the Medical Centre when implementing the Revenue Development Project and services for a fee?**		
Medical research, scientific papers, providing scientific consulting services to companies, individuals, and various sectors of society	49	48.5
Scientific conferences and training courses	52	51.4
Clinical examination, laboratory and radiology examinations in joint projects with other sectors	57	56.4
Investing in unused spaces for a fee	52	51.4
Virtual clinics for all medical clinics	58	57.4
**What is the extent of the expected improvement in the medical services provided when implementing the Revenue Development Project (paid treatment)? 1 is very low, 5 is very high**		
1	7	6.9
2	9	8.9
3	23	22.8
4	37	36.6
5	25	24.8

**Table 4:** Staff suggestions.

Variables	N
**Staff suggestions and comments around developing work in the medical center**	
Infrastructure development	23
Improving the IT network in medical administration, assigning a qualified employee for an information technology unit	8
Increase the number of qualified workforce and provide training programs for all levels.	25
Improvement of logistics and supply services	21
To improve the organization within the center and improvement of the authority matrix and distribution of roles and responsibilities.	6
Developing applicable systems and programs	11
**Managing budget and optimizing it to meet needs**	8
**What investment opportunities can the Medical Services Administration take advantage of to increase its revenues?**	
Laboratory services such as premarital examination, driving licenses, and examination of employees for getting new jobs	14
Training courses and scientific conferences	14
Vaccination programs	1
Investing in unused spaces for a fee	7
Deal with insurance companies to provide service to insured patients	6
Invest in virtual clinics	10
**What challenges may the Medical Centre administration witness if implementing the revenue development project?**	
Lack of demand, as the medical services provided need improvement	5
Medical services are not ready and will not meet the customer’s desires (patient) regarding infrastructure and current medical devices, and the health information program currently used	24
**lack of human workforce**	9
Resistance to change	2
Lack of budget to meet the necessary needs	23

## Data Availability

The data are available from the corresponding author, upon a reasonable request.
